# A Pilot Three Arm Randomised Controlled Trial and Qualitative Study of Extracorporeal Shockwave Therapy for Diabetic Foot Ulcer Healing (SOLEFUL): A Study Protocol

**DOI:** 10.1111/iwj.70176

**Published:** 2025-03-30

**Authors:** L. Hitchman, C. Iglesias, D. Russell, G. Smith, M. Twiddy, I. C. Chetter

**Affiliations:** ^1^ Faculty of Clinical Sciences Hull York Medical School Hull UK; ^2^ Department of Health Sciences University of York York England; ^3^ Leeds Vascular Institute, Teaching Hospitals NHS Trust Leeds UK; ^4^ Leeds Institute of Clinical Trials Research, University of Leeds Leeds UK; ^5^ Hull University Teaching Hospitals NHS Trust Hull UK; ^6^ Institute of Clinical and Applied Sciences, Hull York Medical School Hull UK

**Keywords:** diabetic foot ulcers, extracorporeal shockwave therapy, feasibility studies, randomised controlled trial, wound healing

## Abstract

The development of effective interventions for diabetes‐related foot ulcers (DFU) healing is vital. This protocol outlines a pilot trial and qualitative study investigating ESWT in DFU healing. A pilot three arm placebo controlled double‐blinded randomised controlled trial. Participants with a DFU will be randomised to high dose (500 shocks/cm^2^), low dose (100 shocks/cm^2^) or sham ESWT, in addition to standard care. The primary outcome will be deliverability of a definitive trial. Secondary outcomes are ulcer healing, quality‐of‐life and healthcare resource use at 6 months. The target sample size is 90 participants. The study is registered on clinicaltrials.gov, reference: NCT05380544 and has ethical approval (REC reference: 22/WA/0089). The qualitative interview study will recruit participants who complete, drop‐out and decline to participate in the pilot trial, and healthcare professionals who deliver DFU care. Maximum variable sampling will be used to recruit participants. Data will be analysed with an inductive exploratory approach using reflexive thematic analysis. The pilot trial will ensure methods used will address the research question in the definitive trial. The qualitative study will explore how patients and clinicians interact with the trial to understand the pilot trial findings.

**Summary**

- The study was co‐designed with patients and the public.
- The pilot trial will determine deliverability of a definitive trial of ESWT for DFU healing.
- The qualitative study will explore reasons for engagement and disengagement from participants with the trial and clinicians' attitudes to DFU research.
- Results will enable trial processes to be adapted for the definitive trial to ensure the trial answers whether ESWT is clinically and cost effective for DFU healing.
- A limitation is that this is single centre study in England and results may not translate into other healthcare settings.

## 1 Background

Diabetes‐related foot ulcers (DFUs) are common. Currently 537 million people worldwide are living with diabetes, of which 10% have a DFU [1, 2, 3, 4]. DFUs are associated with poor healing and recurrent infections resulting in multiple hospital admissions, lower limb amputations and death [5, 6, 7, 8, 9]. Prolonged healing and the sequalae of DFUs have a devasting impact on patients' quality of life [10, 11, 12]. The burden of DFUs is also felt by healthcare systems. DFU care costs an estimated £1 billion per year in England and between $11 billion and $17 billion in the United States [13, 14].

The development of effective interventions to aid DFU healing is crucial. National and international guidelines recognise the low quality of evidence underpinning many DFU treatments and call for further research [15, 16]. Areas requiring specific attention are the clinical and cost effectiveness of dressings and other topical treatments. Sparse and poor‐quality evidence for DFU treatments is potentially restricting patients access to effective interventions, exposing patients to ineffective practises and wasting healthcare resources.

Extracorporeal shockwave therapy (ESWT) is a non‐invasive and easily tolerated topical adjunct for DFU healing [17]. It is proposed to improve healing through activation of angiogenesis, cellular proliferation and modulation of the local microbiome [18, 19, 20, 21, 22, 23, 24]. There is evolving evidence that ESWT may be a clinically effective treatment to aid DFU healing, however the existing evidence is low quality and the optimal dosing for DFUs is unknown [25].

This protocol describes a pilot three arm randomised controlled trial (RCT) of ESWT for DFU healing and a qualitative study. The aim of the pilot RCT is to test the deliverability of the proposed trial design before expansion to a fully powered RCT. The qualitative study aims to understand factors that influence participants' engagement in the pilot trial and how clinicians' views and prior experiences impact their engagement in DFU research.

## 2 Methods and Analysis

The pilot RCT protocol is reported with reference to the Standard Protocol Items: Recommendations for Interventional Trials (SPIRIT) and the Consolidated Standards of Reporting Trials (CONSORT) 2010 statement: extension to randomised pilot and feasibility studies [26, 27](Appendix S1). The qualitative study design is reported with reference to the COnsolidated criteria for REporting Qualitative research (COREQ) checklist [28](Appendix S1). The studies have been designed with input from a dedicated patient and public involvement (PPI) group, who have experience of DFUs.

### 2.1 Objectives

The primary objective of the pilot RCT is to determine the deliverability of the proposed definitive trial. The secondary objectives are to explore ulcer‐related outcomes, quality of life and healthcare resource use.

The objective of the qualitative study is to understand why participants engage or disengage with the pilot trial and factors that influence clinician attitudes to DFU research.

### 2.2 Study Design

A pilot three arm placebo controlled double‐blinded randomised controlled trial and qualitative study nested in the pilot trial (patients) and parallel to the pilot trial (clinicians).

### 2.3 Study Setting

A single centre study based in a tertiary care hospital in the United Kingdom (UK). The pilot trial will be conducted in the outpatient setting. Recruited patients (inpatients, outpatients and community patients) will receive the intervention in an outpatient clinic. The qualitative study will be conducted in a private location chosen by the participant and can include the outpatient clinic, a quiet room on the ward, a research office (clinicians only), the university research space, the patient's home, over the telephone or via a videocall.

### 2.4 Eligibility Criteria

Patients with an active DFU below the medial malleolus, present for 4 weeks or longer will be eligible to take part in the pilot trial (Table 1). This includes patients with wounds secondary to surgical debridement and minor amputations and DFU infection, without osteomyelitis. Screening will be performed by the research team in collaboration with the members of the DFU multidisciplinary team (MDT).

**TABLE 1:** Eligibility criteria.

Inclusion criteria	Exclusion criteria
DFU present for ≥ 4 weeks	Interdigital DFU (as the ESWT paddle does not fit between the digits)
Absolute toe pressure ≥ 50 mmHg or an ankle brachial pressure index ≥ 0.7	Diagnosis of malignancy in the treatment area or disseminated, haematological or lymphatic malignancy (contraindication to ESWT)
Capacity to consent	Pregnancy, trying to conceive or breast feeding (contraindication to ESWT)
Willing to receive any of the trial interventions	Active participants in other wound/DFU related trial
Willing to have their DFU photographed	Osteomyelitis^a^
Willing to comply with the follow up schedule	
	Anticoagulation

Abbreviation: DFU, diabetic foot ulcer.

^a^ Clinical suspicion, receiving treatment or proven (radiological or microbiology sample) osteomyelitis.

Participants who complete, drop‐out or decline trial participation will be eligible to take part in the qualitative study. Clinicians nationally who are a member of the DFU MDT will be eligible to participate.

### 2.5 Interventions

The pilot RCT will compare high dose, low dose and sham ESWT. High dose ESWT is 500 shocks/cm^2^ and low dose ESWT is 100 shocks/cm^2^. A minimum of 500 shocks will be administered in the high dose arm and a minimum of 100 shocks will be delivered in the low dose arm. The number of shocks in the active arms is based on the current literature [25].

ESWT will be delivered using a PiezoWave^2^ device (Richard Wolfe, Germany) by a trained researcher. The machine will deliver shockwaves at 0.1 mJ/mm^2^ at 5 Hz and penetrate 5 mm. The DFU will be undressed, cleansed with Prontosan(B Braun, UK) and sharp debrided if required (local standard of care). The DFU will then be covered with a clear film and ultrasound gel applied to the ESWT paddle. Care will be made to ensure there are no air bubbles between the ESWT paddle and film and the ulcer bed.

Sham ESWT will be simulated using a 6 min and 30 s audio‐recording of ESWT. A speaker with the audio‐recording of ESWT will be attached to the top of the ESWT machine. The DFU and ESWT machine will be prepared in the same way as the active arms. Instead of pressing the button to begin delivering shockwaves, the play button on the speaker will be pressed to start the recording. The ESWT paddle will be held onto the ulcer for the duration of the recording. This has been a successful method of blinding in other RCTs of ESWT [29].

High dose, low dose and sham ESWT will be delivered three times over a 7 ± 2 days period. This time interval was chosen to coincide with the standard practice of a dressing change once every 3 days. The treatment will be stopped if the patient is unable to tolerate ESWT.

The decision to investigate the dose of ESWT based on varying the number of shockwaves per cm^2^, as opposed to the frequency of sessions, was on the advice of the PPI group. The PPI group felt longer but fewer sessions of ESWT was more acceptable while the effectiveness of ESWT remains unclear. This is because people with DFUs tend to have many healthcare appointments and increasing the burden of appointments may deter some patients from partaking. In addition, a recent systematic review did not find any differences in healing times between number of ESWT sessions or number of shockwaves delivered [25].

All participants will receive standard care outlined by the NICE and IWGDF guidance [15, 16, 30, 31]. This will include regular dressing changes, sharp debridement, offloading footwear, control of infection and optimisation of blood glucose. Specific aspects of standard care participants receive will be reported. There will be no restrictions on concomitant care or interventions.

### 2.6 Outcomes

The primary outcome of the pilot trial is cohesiveness of the trial components. This will be judged by the eligibility ratio (the number of patients eligible/number of patients screened), recruitment ratio (the number of participants recruited/number of eligible patients), adherence to treatment (number of participants who complete three ESWT sessions/number of participants in the trial), adherence to follow‐up (number of participants who attend all follow‐up visits/number of participants in the trial), and percentage of missing data at each follow‐up point and overall.

Secondary outcomes of the pilot RCT include ulcer‐related outcomes (time to healing, proportion of DFU healed and reduction in DFU size), quality of life, adverse events and healthcare resource use.

### 2.7 Participant Timeline

**FIGURE 1:**
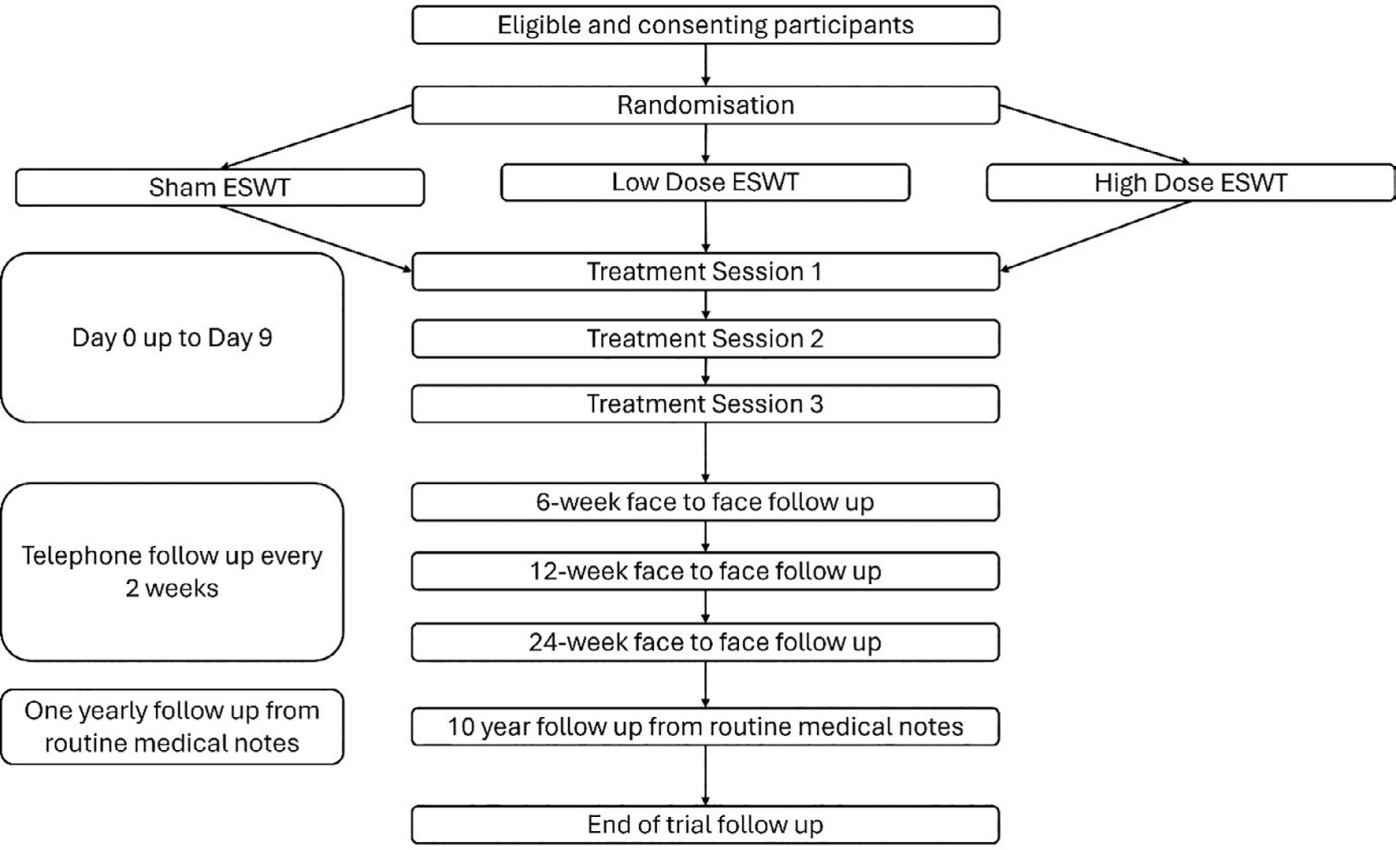
Participant timeline; ESWT: extracorporeal shockwave therapy.

The pilot RCT flow chart is illustrated in Figure 1. At the first visit participant eligibility will be confirmed, informed consent taken by the research doctor, and the participant will be randomised. The three trial interventions will be delivered within the subsequent 7 ± 2 days. Face‐to‐face (F2F) follow‐up will take place at 6, 12 and 24‐weeks post randomisation. Remote follow‐up from electronic medical notes at 1 and 10 years post randomisation.

Participants who complete the F2F follow‐up will be invited to take part in the qualitative study following the 24‐week follow‐up visit. Patients who decline to take part in the trial or drop out of the trial will be invited to take part in the qualitative study at the point of declining/dropping out and be interviewed at the earliest convenient time to them. Clinicians will be interviewed at a time convenient to them.

### 2.8 Sample Size

The pilot RCT aims to recruit 90 participants overall. This figure is based on the minimum recommended number of trial participants required to estimate the standard deviation to inform a sample size calculation for a definitive trial [32, 33, 34, 35, 36].

Sample size calculation: 23*3 = 69 minimum number of participants*number of arms.

69*0.3% = 21 30% estimated attrition.

69 + 21 = 90.

The qualitative study sample size is informed by informational power, whereby the more information a sample holds, the smaller the sample needs to be [37, 38]. The aim of this study is narrow and each participant will hold experience relevant to the aim of the study. The researcher undertaking the interviews has experience in DFU, treatments and prior experience of interviewing. The combination of patient and researcher experience is likely to result in more in‐depth data exploration, resulting in fewer participants needed. The qualitative study aims to recruit 36 participants in total split between the following groups:

– Complete the trial: 12
– Drop‐out of the trip: 6
– Decline trial participation: 6
– DFU MDT clinicians: 12

### 2.9 Recruitment

All pilot RCT participants will be recruited from one tertiary care hospital from the inpatient and outpatient services, with links to the community diabetic foot services. The National Institute of Health and Social Care Research (NIHR) Clinical Research Network will also support recruitment to the study through their contacts with local general practice and community services (study reference CPMS: 52540). The trial will recruit patients over an 18 months recruitment period.

Potential participants will be approached by the research team after discussion with the clinical team and provided a patient information sheet (PIS). This will be followed by a telephone call within the subsequent 7 days to allow patients time to read and understand the information. If a researcher is not available, the clinician can provide the patient with a PIS and complete a consent to contact form.

A log of all screened patients will be kept to document reasons screened patients were not eligible and eligible patients did not enter the study. This will be used to monitor for under‐representation of under‐served groups and identify barriers to entering the trial.

Qualitative study participants will be recruited from the same patient pool as the pilot RCT participants. Purposeful sampling will be used to recruit participants with varying demographics (gender, age, ethnicity), treatment allocation and wound severity (based on Wound Ischaemia, foot Infection stage). Patients will either be approached at a F2F appointment or invited via the telephone. Clinicians will be recruited nationally using purposeful sampling to ensure variation based on the healthcare professional group, location in the United Kingdom and type of healthcare setting. They will be identified at national conferences, through the Vascular Society Diabetic Foot Specialist Interest Group, X (formally Twitter) and snowballing. Clinicians will be approached in person or via email. All potential participants will be provided with a PIS. A log will be kept of all screened patients and clinicians to record reasons they were ineligible or declined participation.

### 2.10 Allocation

Participants will be randomised on a 1:1:1 allocation ratio to sham ESWT, low dose ESWT and high dose ESWT. Participants will be stratified by ulcer size (< 1 and ≥ 1 cm^2^) and ischaemia (ABPI 0.7–0.79 and ≥ 0.8; OR absolute toe pressure 50–59 and ≥ 60 mmHg). These two factors are predictive of healing [39, 40]. Stratified block randomisation with randomly varying block sizes of 3 and 6 will be used. Randomisation will be performed using an online randomised tool hosted at York Randomisation Service, York Trials Unit. The site researcher will enter the participants details into the online tool. The York Trials Unit trial manager, Principal Investigator and the site researcher will then receive an email with the participant's trial arm allocation.

### 2.11 Blinding

Trial participants will be blinded to treatment allocation. ESWT is not routinely used in DFU care and therefore it is highly unlikely that participants will have experienced ESWT before and be able to identify the sham. In addition, many of the participants will have peripheral neuropathy so will not be able to feel any sensations associated with ESWT.

The outcome assessors, who are experts in wound care, will assess for DFU healing. They will be blinded to the participants treatment allocation. The outcome assessors will not be involved in randomisation or delivery of ESWT. Unblinding will be permissible if there is an adverse event related to the trial intervention. This will be achieved by contacting the Principal Investigator who will determine if the event was related to the intervention and, if so, will inform the clinical team and patient of the arm allocation. Participants can be unblinded following the 24‐week F2F follow‐up visit if they chose.

### 2.12 Data Collection

Data items for collection are based on international reporting standards for DFU studies [41]. Data will be collected from the patient, electronic patient records or direct patient measurements and entered onto a paper case report form (CRF) (Table 2).

**TABLE 2:** Sources of data.

	Patient reported	Medical records	Direct measurements/observation
Enrolment			
Eligibility log			X
Recruitment log			X
Informed consent			X
Allocation			X
Deliverability			
Adherence to treatment			X
Adherence to follow up			X
Intervention			
Treatment completed			X
Number of shockwaves			X
Time to deliver ESWT			X
Side effects of ESWT	X		X
Demographics			
Age	X		
Sex	X		
Ethnicity	X		
BMI	X	X	X
Smoking status	X		
Weekly alcohol intake	X		
Type of diabetes mellitus	X	X	
HbA1c		X	
Diabetes management	X	X	
Cardiovascular disease	X	X	
Respiratory disease	X	X	
Chronic kidney disease	X	X	
Surgical history	X	X	
Allergies	X	X	
Medications	X	X	
Antibiotic use	X	X	
Ulcer characteristics			
Site			X
Laterality			X
Ulcer age	X	X	
SINBAD score			X
WIfI stage			X
Foot deformity			X
DFU photograph			X
Area			X
Depth			X
ABPI			X
Absolute toe pressure			X
Time to assess DFU			X
Ulcer care			
Dressing(s) type	X	X	X
Offloading footwear	X	X	X
Sharp debridement	X	X	X
Other interventions	X	X	X
Quality of life			
EQ‐5D‐3L	X		
DFS‐SF	X		
Wound‐Qol‐14	X		
Healthcare use^a^			
Number of outpatient appointments	X	X	
Number of GP appointments	X	X	
Number of hospital admissions	X	X	
Length of stay	X	X	
Number of practice nurse appointments	X	X	
Number of district nurse appointments	X	X	
Number of podiatrist appointments	X	X	
Number of prescriptions	X	X	
Number of operations	X	X	
Ulcer healing			X
Adverse events	X	X	X
Qualitative interview	X		

Abbreviations: ABPI: ankle brachial pressure index; BMI: body mass index; DFS‐SF: diabetic foot ulcer scale—short form; DFU: diabetic foot ulcer; EQ5D5L: EuroQol 5‐dimensions 5‐levels; ESWT: extracorporeal shockwave therapy; GP: general practitioner; INBAD: site ischaemia neuropathy bacterial infection area and depth score; WIfI: wound ischaemia foot infection stage.

^a^ Taken from the patient healthcare resource use diary.

Data relating to participant demographics, the DFU, standard care and quality of life will be taken at baseline.

Data relating to the number of shockwaves delivered, time to deliver ESWT and side effects will be recorded at each intervention session.

DFU measurements, standard care, quality of life and health resource use will be measured after the third treatment and at 6‐, 12‐ and 24‐weeks post randomisation (Table 3). DFU will be measured using tape measure and probe at the longest axis in each dimension. Data on healing, recurrence, major lower limb amputation and death will be collected until 1 year post randomisation. Healing is defined as complete re‐epithelisation without eschar assessed 2 weeks apart by a blinded wound assessor. Recurrence is defined as a break in the epithelium at the same site of the DFU after healing has been confirmed.

**TABLE 3:** Schedule of enrolment, interventions and assessments.

	Enrolment	Allocation	Post allocation								
			ESWT treatment session (high/low/placebo; session)			Face‐to‐ face follow‐up (weeks post randomisation)			Telephone follow up^a^ (weeks post randomisation)	DFU healed visit^b^ (weeks)	Follow up from routine medical records (years)
Time point	−1	0	1	2	3	6	12	24	2–24	1–24	1–10
Enrolment											
Eligibility log	X										
Recruitment log	X										
Informed consent	X										
Allocation		X									
Deliverability											
Adherence to treatment			X	X	X						
Adherence to follow up						X	X	X	X	X	
Intervention											
Treatment completed			X	X	X						
Number of shockwaves			X	X	X						
Time to deliver ESWT			X	X	X						
Side effects of ESWT			X	X	X						
Assessments											
Demographics											
Age			X								
Sex			X								
Ethnicity			X								
BMI			X								
Smoking status			X								
Weekly alcohol intake			X								
Type of diabetes mellitus			X								
HbA1c			X								
Diabetes management			X		X	X	X	X			
Cardiovascular disease			X								
Respiratory disease			X								
Chronic kidney disease			X								
Surgical history			X								
Allergies			X								
Medications			X								
Antibiotic use			X		X	X	X	X			
Ulcer characteristics											
Site			X								
Laterality			X								
Ulcer age			X								
SINBAD score			X								
WIfI stage			X		X	X	X	X			
Foot deformity			X								
DFU photograph			X		X	X	X	X		X	
Area			X	X	X	X	X	X			
Depth			X		X	X	X				
ABPI			X								
Absolute toe pressure			X								
Time to assess DFU			X								
Ulcer care											
Dressing(s) type			X		X	X	X	X			
Offloading footwear			X		X	X	X	X			
Sharp debridement			X		X	X	X	X			
Other interventions			X		X	X	X	X			
Quality of life											
EQ‐5D‐3L			X		X	X	X	X			
DFS‐SF			X		X	X	X	X			
Wound‐Qol‐14			X		X	X	X	X			
Health resource use											
Number of outpatient appointments					X	X	X	X			
Number of GP appointments					X	X	X	X			
Number of hospital admissions					X	X	X	X			
Length of stay					X	X	X	X			
Number of practice nurse appointments					X	X	X	X			
Number of district nurse appointments					X	X	X	X			
Number of podiatrist appointments					X	X	X	X			
Number of prescriptions					X	X	X	X			
Number of operations					X	X	X	X			
Ulcer healing					X	X	X	X	X	X	X
Adverse events			X	X	X	X	X	X	X	X	X
Qualitative interview	X^c^			X^d^	X^d^	X^d^	X^d^	X	X^d^	X^d^	

Abbreviations: ABPI: ankle brachial pressure index; BMI: body mass index; DFS‐SF: diabetic foot ulcer scale – short form; DFU: diabetic foot ulcer; EQ5D5L: EuroQol 5‐dimensions 5‐levels; ESWT: extracorporeal shockwave therapy; GP: general practitioner; SINBAD: site ischaemia neuropathy bacterial infection area and depth score; WIfI: wound ischaemia foot infection stage.

^a^ Every 2 weeks between finishing ESWT and the 24‐week follow‐up visit.

^b^ If the patient reports the ulcer is healed, repeated 2 weeks later to ensure the DFU remains healed.

^c^ If the eligible patient declines trial participation.

^d^ If the participant decides to withdraw from the trial before the 24‐week follow‐up visit.

Health‐related quality of life will be measured using EuroQol 5D‐5L (EQ‐5D‐5L), diabetic foot ulcer scale short form (DFS‐SF) and Wound‐Qol‐14. EQ‐5D‐5L will be used to aid economic evaluation of ESWT [42, 43, 44]. DFS‐SF is a disease‐specific validated tool that measures health‐related quality of life in participants with a DFU [45]. Wound‐Qol‐14 will be validated during the pilot RCT and is hypothesised to have a higher completion rate than DFS‐SF as it is shorter (14 items versus 29 items). The tool has been validated in a population with chronic wounds, which included patients with DFUs [46, 47].

Participants can withdraw from the intervention, F2F follow‐up, telephone follow‐up or completely withdraw from the trial. Consent will be sought to use of the data already collected and to continue collecting data from routine medical records. If a patient is lost to follow‐up, data will be collected from routine medical records.

For the qualitative study, data will be collected using semi‐structured in‐depth interviews guided by group specific topic guides, informed by previous research and clinical experience. Interviews will be conducted by the lead author, who is a female vascular surgery registrar with training in qualitative interviewing. The topic guides will contain a mix of open and focused questions and will be piloted prior to use. All interviews will be audio‐recorded using a digital voice recorder and transcribed verbatim. Transcription will be undertaken by the researcher for the first three interviews. Microsoft Office Word transcribe tool will be used to transcribe the subsequent recordings.

### 2.13 Data Management

Data will be collected onto paper CRFs by a research team member named on trial delegation log. Completed CRFs will be sent next day, tracked and signed for via Royal Mail to York Trials Unit, based at the University of York. No patient identifiable information will be mailed. Data will be entered into a secure REDCap database hosted on the University of York cloud‐based server by a data imputer and checked by a second data imputer. All data queries will be raised with the research team.

The paper trial file, incomplete CRFs and participant consent forms will be stored in locked filing cabinets in the research office at Hull University Teaching Hospitals NHS Trust, accessible only by swipe card access.

Digital data, including screening logs, audio recordings, patient letters, transcripts and DFU photographs will be stored in the electronic trial file only accessible by the trial researchers. The digital trial file will be held on the NHS Trust server, which is secured with firewalls and antivirus software. Only researchers on the delegation log will have access to the electronic trial file.

### 2.14 Statistical Analysis

Eligibility and recruitment rates will be reported for the entire population screened. Intervention adherence, crossover, follow‐up and missing data will be reported for each arm and in total. The progression of participants through the trial will be illustrated with a flow diagram. Reasons for ineligibility, non‐consent, non‐compliance and drop‐out will be reported.

Baseline characteristics will be summarised by arm and in total for randomised participants. Continuous outcomes will be reported as means, standard deviations, medians and interquartile ranges. Categorical data will be reported as counts and percentages.

Secondary outcomes will be reported descriptively at each follow‐up time point for each trial arm. No statistical testing will be undertaken as this is a pilot trial, however between group differences will be presented with associated confidence intervals and illustrated in appropriate graphs (e.g., Kaplan–Meier plots). All comparisons will follow intention‐to‐treat principles.

The number of adverse events will be reported by type and severity for each arm and overall. Events will be summarised as total event rate and mean/median number of events per patient in each arm.

Data will be analysed using Microsoft Excel (2021; WA, USA), IMB SPSS (version 28.0.0.0; Armonk, NY, USA) and STATA (Version 18; TX, USA).

### 2.15 Qualitative Analysis

Audio‐recordings will be listened back to, and transcripts will be read twice for data familiarisation. An inductive exploratory approach and reflexive thematic analysis will be used to analyse data [48, 49]. Throughout the data collection and analysis processes a reflexive diary and memos will be kept to document key decisions made and interpretation of data. Peer briefings will take place between the lead researcher (LH) and the senior qualitative researcher (MT). Data from the trial participants, trial decliners and clinicians will be analysed separately, and themes will then be compared. Transcripts will be uploaded onto Nvivo (Version 14; Denver, CO, USA) for coding and analysis.

### 2.16 Data Monitoring

Eligibility rates, recruitment rates, treatment adherence, follow‐up adherence and adverse events will be reviewed by the trial management group (TMG) monthly and the trial steering committee (TSC) bi‐annually. The TSC will use Stop/Go criteria to decide whether to continue the pilot trial or pause recruitment and amend trial processes. The Stop/Go criteria [50] are:

– Green (continue): > 60% recruitment, adherence to treatment arm and follow‐up attendance per month.
– Amber (continue, review trial processes): 30%–60% recruitment, adherence to treatment arm and follow‐up attendance per month.
– Red
  ○ < 30% (Stop, amend trial processes) recruitment, adherence to treatment arm and follow‐up attendance per month.
  ○ Serious adverse events related to ESWT (stop, review).

The auditing of the trial processes will take place by independent auditors from the Sponsor's Research, Development and Innovation (RD&I) Department.

### 2.17 Harms

All side effects will be recorded during the pilot RCT. The expected transient side effects of ESWT are mild pain, burning sensation, itchiness and erythema around the ulcer. The standard definitions of adverse event (AE), serious adverse event (SAE), unexpected and related will be used [51]. All AEs and device failures will be recorded. All SAEs will be reported to the Principal Investigator (PI) within 24 h of the event occurring. The PI will report SAEs to the Sponsor and Research Ethics Committee (REC), following Health Research Authority (HRA) guidance.

## 3 Ethics and Dissemination

### 3.1 Research Ethics Approval

Ethical approval for the study was gained from the NHS Health Research Authority (HRA) (IRAS: 311664; REC reference: 22/WA/0089; Protocol v2.3, 11/05/2023). The study is sponsored by Hull University Teaching Hospitals NHS Trust. The study will be undertaken in accordance with Declaration of Helsinki (2008) [52], and all participants will provide written informed consent prior to engaging in study activities. Data will be managed in line with GDPR 2018 guidance. The study is registered on clinicaltrials.gov (NCT05380544).

### 3.2 Protocol Amendments

Protocol amendments will be submitted to the HRA via the Integrated Research Application System (IRAS) amendment tool following approval from the Sponsor's RD&I manager. All amended documents will be superseded in both the electronic and paper trial file.

### 3.3 Consent

All participants will be adults and must have capacity to give written informed consent. Consent will be taken by named researcher doctors on the delegation log. Consent will be recorded on a paper consent form which must be signed and dated by the participant and the research doctor.

### 3.4 Declaration of Interests

The trial is funded by the NIHR/Diabetes UK Doctoral Research Fellowship Grant (L.H.) (NIHR301807). There are no other competing interests.

### 3.5 Access to Data

The Chief Investigator (IC) and PI (LH) will have access to the data with patient identifiers. The statisticians at York Trials Unit will have access to anonymised pilot RCT data.

### 3.6 Dissemination Policy

The trial will be reported in line with the CONSORT guidance [26] and qualitative study will be reported with reference to the COREQ checklist [28]. Healthcare professionals will be informed of the trial results through conferences proceedings and publication in a peer review journal. Authors meeting the International Committee of Medical Journal Editors definition of an author will be included in any publication [53]. Trial participants will receive a lay summary of the trial findings. The public will be informed of the trial findings through dissemination of a lay summary in a multimedia format in collaboration with Vascular Research UK, Diabetes UK and Circulation Foundation.

## 4 Discussion

This protocol outlines the study design for a pilot RCT evaluating ESWT for DFU healing and a qualitative study. Findings from a recent systematic review reported promising results with ESWT for DFU healing, but low certainty in evidence on the effectiveness and unclear impact of dosing restricted recommendations that ESWT should become part of routine care [25]. Certainty in the data was often downgraded due to imprecision, indirectness and risk of bias. Specific areas of concern were the randomisation process, (non‐)blinding of participants and outcomes assessors, and outcome reporting. This pilot RCT aims to address these areas and ensure methods used will provide reliable results in a definitive RCT.

The NIHR view pilot RCTs as value for money when they answer key uncertainties in trial conduct [54]. In this trial, relevant uncertainties include number of eligible patients, patient willingness to participate, adherence to the treatment arms and whether it is possible to collect the relevant outcome data using the methods described. Identifying problems with recruitment, delivery of the intervention and collection of outcomes measures in the pilot RCT allows for these processes to be refined for the definitive trial and minimises the chances of wasting research resources by proceeding straight to a definitive trial that is not fit for purpose. The failure of trial methods in this pilot RCT is an outcome, not a detriment to the evidence generated. In addition, knowledge of the expected numbers of participants to engage allows for logistics to be planned, for example how many trial sites will be needed and the expected time to recruit the sample size. This will inform the funding application for a definitive trial.

When compared to other trials of ESWT in DFU, the eligibility criteria in this pilot RCT slightly differs as it includes participants with neuro‐ischaemic and infected DFUs [55, 56, 57, 58, 59, 60]. The rationale in ESWT is hypothesised to stimulate angiogenesis and increase eradication of bacteria in the presence of antibiotics. Including these participants in the pilot RCT will explore whether ESWT is safe (judged by numbers of SAEs) and number of participants with these ulcer characteristics. This will inform statistical and sample size planning in the definitive trial to explore the effectiveness of ESWT in these patient groups.

Three treatment arms are included in this pilot RCT to explore the dosing of ESWT. Currently, there does not appear to be a relationship between the number of shocks delivered or the number of sessions of ESWT and DFU healing outcomes [25]. While this is possibly due to low quality data, the dosing of ESWT does necessitate exploration if it is to be used in clinical practice. The amount of resources used and associated clinical outcomes will influence the cost effectiveness of ESWT and likelihood of implementation.

The qualitative study will aid understanding of the quantitative results of the study. Reviews of mixed method RCTs found how participants understand the trial process, perceive risk and benefits of treatment options and trust in healthcare professionals influence their decision to participate [61]. This study will explore which specific factors in this pilot trial are influencing participants engagement, with reference to these overarching themes. This will help tackle issues with recruitment and retention often experienced by multicentre RCTs [62].

The PPI group were crucial to designing the trial process. As researchers with no experience of having a DFU, understanding the burden of the proposed trial processes was vital in designing a trial fit for participants. The PPI group were instrumental in deciding how ESWT dosing should be investigated and the follow‐up approach. The trial will continue to involve the PPI members in the TSC meetings.

There will be limitations of the pilot RCT. Data collected will be reflective of a single centre in the United Kingdom, which could mean results are not reproductible in other centres. NICE NG19 guideline outlines the structure DFU services should conform to in the United Kingdom, and in 2021, 91% of NHS Trusts reported they were adherent to this guideline, although there could be inaccuracies as adherence is self‐reported [9]. In centres who deliver care using a similar model, the trial findings should be reproducible. Other sources of variation could include differing local population demographics, local diabetic foot MDT research experience and local research delivery infrastructure.

This pilot RCT will inform a definitive trial evaluating the role of ESWT in DFU care. It will provide data to demonstrate the proposed trial is deliverable and can evaluate the effectiveness of ESWT in DFU healing to advance DFU care.

## Conflicts of Interest

The authors declare no conflicts of interest.

## Supporting information


Appendix S1.

## Data Availability

Requests to access the study data can be made in writing to the lead and senior author.
