# Supplementary material for: A Pilot Three Arm Randomised Controlled Trial and Qualitative Study of Extracorporeal Shockwave Therapy for Diabetic Foot Ulcer Healing (SOLEFUL): A Study Protocol
Source: Int Wound J. 2025 Mar 30;22(4):e70176. doi: 10.1111/iwj.70176 (PMC11955253; doi:10.1111/iwj.70176)
Supplement: Supplementary file 1 — Appendix S1. [file IWJ-22-e70176-s001.zip › IWJ70176-sup-0001-AppendixS1/Fillable-SPIRIT-Outcomes-2022-Checklist-with-SPIRIT-2013 SOLEFUL.doc]

**^SPIRIT-Outcomes 2022 Checklist (for combined completion of SPIRIT 2013 and SPIRIT-Outcomes 2022 items)^**a

|  | **Section** |  | **Item** |  | **SPIRIT 2013 Item** |  |  | **SPIRIT-Outcomes 2022 item** |  | **Location** |
| --- | --- | --- | --- | --- | --- | --- | --- | --- | --- | --- |
|  |  |  | **No.** |  |  |  |  |  |  | **Reported^b^** |
|  |  |  |  |  |  |  |  |  |  |  |
|  | **Administrative** | | **information** |  |  |  |  |  |  |  |
|  |  |  |  |  |  |  |  |  |  |  |
|  | Title | | 1 |  | Descriptive title identifying the | | - | |  |  |
|  |  |  |  |  | study design, population, | |  |  |  | Page 1 |
|  |  |  |  |  | interventions, and, if applicable, | |  |  |  |  |
|  |  |  |  |  |  |  |  |  |  |  |
|  |  |  |  |  | trial acronym | |  |  |  |  |
|  | Trial registration | | 2a |  | Trial identifier and registry name. | | - | |  |  |
|  |  |  |  |  | If not yet registered, name of | |  |  |  | Page 2 |
|  |  |  |  |  | intended registry | |  |  |  |  |
|  |  |  | 2b |  | All items from the World Health | | - | |  |  |
|  |  |  |  |  | Organization Trial Registration | |  |  |  | Page 2 |
|  |  |  |  |  | Data Set | |  |  |  |  |
|  | Protocol version | | 3 |  | Date and version identifier | | - | |  |  |
|  |  |  |  |  |  |  |  |  |  | Page 11 |
|  |  | |  |  |  | |  | |  |  |
|  | Funding | | 4 |  | Sources and types of financial, | | - | |  |  |
|  |  |  |  |  | material, and other support | |  |  |  | Page 14 |
|  |  | |  |  |  | |  | |  |  |
|  | Roles and | | 5a |  | Names, affiliations, and roles of | | - | |  |  |
|  | responsibilities | |  |  | protocol contributors | |  |  |  | Page 1, 14 |
|  |  |  |  |  |  | |  | |  |  |
|  |  |  | 5b |  | Name and contact information for | | - | |  |  |
|  |  |  |  |  | the trial sponsor | |  |  |  | Appendix |
|  |  |  |  |  |  | |  | |  |  |
|  |  |  | 5c |  | Role of study sponsor and | | - | |  | Page 11, 14 |
|  |  |  |  |  | funders, if any, in study design; | |  |  |  |  |
|  |  |  |  |  | collection, management, analysis, | |  |  |  |  |
|  |  |  |  |  | and interpretation of data; writing | |  |  |  |  |
|  |  |  |  |  | of the report; and the decision to | |  |  |  |  |
|  |  |  |  |  | submit the report for publication, | |  |  |  |  |
|  |  |  |  |  | including whether they will have | |  |  |  |  |
|  |  |  |  |  | ultimate authority over any of | |  |  |  |  |
|  |  |  |  |  | these activities | |  |  |  |  |
|  |  |  | 5d |  | Composition, roles, and | | - | |  | Page 11 |
|  |  |  |  |  | responsibilities of the coordinating | |  |  |  |  |
|  |  |  |  |  | centre, steering committee, | |  |  |  |  |
|  |  |  |  |  | endpoint adjudication committee, | |  |  |  |  |
|  |  |  |  |  | data management team, and | |  |  |  |  |
|  |  |  |  |  | other individuals or groups | |  |  |  |  |
|  |  |  |  |  | overseeing the trial, if applicable | |  |  |  |  |
|  |  |  |  |  | (see Item 21a for data monitoring | |  |  |  |  |
|  |  |  |  |  | committee) | |  |  |  |  |
|  | **Introduction** | |  |  |  |  |  |  |  |  |
|  |  |  |  |  |  |  |  |  |  |  |
|  | Background and | | 6a |  | Description of research question | | - | |  | Page 4 |
|  | rationale | |  |  | and justification for undertaking | |  |  |  |  |
|  |  |  |  |  | the trial, including summary of | |  |  |  |  |
|  |  |  |  |  | relevant studies (published and | |  |  |  |  |
|  |  |  |  |  | unpublished) examining benefits | |  |  |  |  |
|  |  |  |  |  | and harms for each intervention | |  |  |  |  |
|  |  |  | 6b |  | Explanation for choice of | | - | |  | Page 6 |
|  |  |  |  |  | comparators | |  |  |  |  |
|  |  |  |  |  |  |  |  |  |  |  |
|  | Objectives | | 7 |  | Specific objectives or hypotheses | | - | |  | Page 5 |
|  |  |  |  |  |  |  |  |  |  |  |
|  |  |  |  |  |  |  |  |  |  |  |

1

|  | **Section** |  | **Item** |  | **SPIRIT 2013 Item** |  |  | **SPIRIT-Outcomes 2022 item** |  | **Location** |
| --- | --- | --- | --- | --- | --- | --- | --- | --- | --- | --- |
|  |  |  | **No.** |  |  |  |  |  |  | **Reported^b^** |
|  |  |  |  |  |  |  |  |  |  |  |
|  | Trial design | | 8 |  | Description of trial design | | - | |  | Page 5 |
|  |  |  |  |  | including type of trial (eg, parallel | |  |  |  |  |
|  |  |  |  |  | group, crossover, factorial, single | |  |  |  |  |
|  |  |  |  |  | group), allocation ratio, and | |  |  |  |  |
|  |  |  |  |  | framework (eg, superiority, | |  |  |  |  |
|  |  |  |  |  | equivalence, noninferiority, | |  |  |  |  |
|  |  |  |  |  | exploratory) | |  |  |  |  |
|  |  | |  |  | | |  |  |  |  |
|  | **Methods: Participants, interventions, and outcomes** | | | | | |  |  |  |  |
|  |  |  |  |  |  |  |  |  |  |  |
|  | Study setting | | 9 |  | Description of study settings (eg, | | - | |  | Page 5 |
|  |  |  |  |  | community clinic, academic | |  |  |  |  |
|  |  |  |  |  | hospital) and list of countries | |  |  |  |  |
|  |  |  |  |  | where data will be collected. | |  |  |  |  |
|  |  |  |  |  | Reference to where list of study | |  |  |  |  |
|  |  |  |  |  | sites can be obtained | |  |  |  |  |
|  | Eligibility criteria | | 10 |  | Inclusion and exclusion criteria for | | - | |  | Page 5 |
|  |  |  |  |  | participants. If applicable, | |  |  |  |  |
|  |  |  |  |  | eligibility criteria for study centres | |  |  |  |  |
|  |  |  |  |  | and individuals who will perform | |  |  |  |  |
|  |  |  |  |  | the interventions (eg, surgeons, | |  |  |  |  |
|  |  |  |  |  | psychotherapists) | |  |  |  |  |
|  | Interventions | | 11a |  | Interventions for each group with | | - | |  | Page 5, 6 |
|  |  |  |  |  | sufficient detail to allow | |  |  |  |  |
|  |  |  |  |  | replication, including how and | |  |  |  |  |
|  |  |  |  |  | when they will be administered | |  |  |  |  |
|  |  |  |  |  | (for specific guidance see TIDieR | |  |  |  |  |
|  |  |  |  |  | checklist and guide) | |  |  |  |  |
|  |  |  | 11b |  | Criteria for discontinuing or | | - | |  | Page 6 |
|  |  |  |  |  | modifying allocated interventions | |  |  |  |  |
|  |  |  |  |  | for a given trial participant (eg, | |  |  |  |  |
|  |  |  |  |  | drug dose change in response to | |  |  |  |  |
|  |  |  |  |  | harms, participant request, or | |  |  |  |  |
|  |  |  |  |  | improving/worsening disease) | |  |  |  |  |
|  |  |  | 11c |  | Strategies to improve adherence | | - | |  | Page 6 |
|  |  |  |  |  | to intervention protocols, and any | |  |  |  |  |
|  |  |  |  |  | procedures for monitoring | |  |  |  |  |
|  |  |  |  |  | adherence (eg, drug tablet return, | |  |  |  |  |
|  |  |  |  |  | laboratory tests) | |  |  |  |  |
|  |  |  | 11d |  | Relevant concomitant care and | | - | |  | Page 6 |
|  |  |  |  |  | interventions that are permitted or | |  |  |  |  |
|  |  |  |  |  | prohibited during the trial | |  |  |  |  |
|  | Outcomes | | 12 |  | Primary, secondary, and other | | - | |  | Page 6, 9, Table |
|  |  |  |  |  | outcomes, including the specific | |  |  |  | 2, Table 3 |
|  |  |  |  |  | measurement variable (eg, | |  |  |  |  |
|  |  |  |  |  | systolic blood pressure), analysis | |  |  |  |  |
|  |  |  |  |  | metric (eg, change from baseline, | |  |  |  |  |
|  |  |  |  |  | final value, time to event), method | |  |  |  |  |
|  |  |  |  |  | of aggregation (eg, median, | |  |  |  |  |
|  |  |  |  |  | proportion), and time point for | |  |  |  |  |
|  |  |  |  |  | each outcome. Explanation of the | |  |  |  |  |
|  |  |  |  |  | clinical relevance of chosen | |  |  |  |  |
|  |  |  |  |  | efficacy and harm outcomes is | |  |  |  |  |
|  |  |  |  |  | strongly recommended | |  |  |  |  |
|  |  |  |  |  |  |  |  |  |  |  |

2

|  | **Section** |  | **Item** |  | **SPIRIT 2013 Item** |  |  | **SPIRIT-Outcomes 2022 item** |  | **Location** |
| --- | --- | --- | --- | --- | --- | --- | --- | --- | --- | --- |
|  |  |  | **No.** |  |  |  |  |  |  | **Reported^b^** |
|  |  |  |  |  |  |  |  |  |  |  |
|  |  |  | 12.1 |  |  |  |  | Provide a rationale for the selection | | Page 4 |
|  |  |  |  |  |  |  |  | of the domain for the trial s primary | |  |
|  |  |  |  |  |  |  |  | outcome | |  |
|  |  |  | 12.2 |  |  |  |  | If the analysis metric for the primary | | NA |
|  |  |  |  |  |  |  |  | outcome represents within-participant | |  |
|  |  |  |  |  |  |  |  | change, define and justify the | |  |
|  |  |  |  |  |  |  |  | minimal important change in | |  |
|  |  |  |  |  |  |  |  | individuals | |  |
|  |  |  | 12.3 |  |  |  | If the outcome data collected are | | | NA |
|  |  |  |  |  |  |  | continuous but will be analyzed as | | |  |
|  |  |  |  |  |  |  | categorical (method of aggregation), | | |  |
|  |  |  |  |  |  |  | specify the cutoff values to be used | | |  |
|  |  |  | 12.4 |  |  |  |  | If outcome assessments will be | | Page 9, |
|  |  |  |  |  |  |  |  | performed at several time points | |  |
|  |  |  |  |  |  |  |  |  |  | Table 2, |
|  |  |  |  |  |  |  |  | after randomization, state the time | |  |
|  |  |  |  |  |  |  |  |  |  | Table 3 |
|  |  |  |  |  |  |  |  | points that will be used for analysis | |  |
|  |  |  |  |  |  |  |  |  |  |  |
|  |  |  | 12.5 |  |  |  |  | If a composite outcome is used, | | NA |
|  |  |  |  |  |  |  |  | define all individual components | |  |
|  |  |  |  |  |  |  |  | of the composite outcome | |  |
|  |  | |  |  |  | |  | |  |  |
|  | Participant | | 13 |  | Time schedule of enrolment, | | - | |  | Figure 1 |
|  | timeline | |  |  | interventions (including any run- | |  |  |  |  |
|  |  |  |  |  | ins and washouts), assessments, | |  |  |  |  |
|  |  |  |  |  | and visits for participants. A | |  |  |  |  |
|  |  |  |  |  | schematic diagram is highly | |  |  |  |  |
|  |  |  |  |  | recommended (see Figure) | |  |  |  |  |
|  | Sample size | | 14 |  | Estimated number of participants | | - | |  | Page 7 |
|  |  |  |  |  | needed to achieve study | |  |  |  |  |
|  |  |  |  |  | objectives and how it was | |  |  |  |  |
|  |  |  |  |  | determined, including clinical and | |  |  |  |  |
|  |  |  |  |  | statistical assumptions supporting | |  |  |  |  |
|  |  |  |  |  | any sample size calculations | |  |  |  |  |
|  |  |  | 14.1 |  |  |  |  | Define and justify the target | | NA |
|  |  |  |  |  |  |  |  | difference between treatment groups | |  |
|  |  |  |  |  |  |  |  | (eg, the minimal important difference) | |  |
|  | Recruitment | | 15 |  | Strategies for achieving adequate | | - | |  | Page 8 |
|  |  |  |  |  | participant enrolment to reach | |  |  |  |  |
|  |  |  |  |  | target sample size | |  |  |  |  |
|  |  | |  |  | | |  |  |  |  |
|  | **Methods: Assignment of interventions (for controlled trials)** | | | | | |  |  |  |  |
|  |  |  |  |  |  |  |  |  |  |  |
|  | Allocation: | |  |  |  |  |  |  |  |  |
|  |  | |  |  |  | |  | |  |  |
|  | Sequence | | 16a |  | Method of generating the | | - | |  | Page 8 |
|  | generation | |  |  | allocation sequence (eg, | |  |  |  |  |
|  |  |  |  |  | computer-generated random | |  |  |  |  |
|  |  |  |  |  | numbers), and list of any factors | |  |  |  |  |
|  |  |  |  |  | for stratification. To reduce | |  |  |  |  |
|  |  |  |  |  | predictability of a random | |  |  |  |  |
|  |  |  |  |  | sequence, details of any planned | |  |  |  |  |
|  |  |  |  |  | restriction (eg, blocking) should | |  |  |  |  |
|  |  |  |  |  | be provided in a separate | |  |  |  |  |
|  |  |  |  |  | document that is unavailable to | |  |  |  |  |
|  |  |  |  |  | those who enrol participants or | |  |  |  |  |
|  |  |  |  |  | assign interventions | |  |  |  |  |

3

|  | **Section** |  | **Item** |  | **SPIRIT 2013 Item** |  |  | **SPIRIT-Outcomes 2022 item** |  | **Location** |
| --- | --- | --- | --- | --- | --- | --- | --- | --- | --- | --- |
|  |  |  | **No.** |  |  |  |  |  |  | **Reported^b^** |
|  |  |  |  |  |  |  |  |  |  |  |
|  | Allocation | | 16b |  | Mechanism of implementing the | | - | |  | Page 8 |
|  | concealment | |  |  | allocation sequence (eg, central | |  |  |  |  |
|  | mechanism | |  |  | telephone; sequentially | |  |  |  |  |
|  |  |  |  |  | numbered, opaque, sealed | |  |  |  |  |
|  |  |  |  |  | envelopes), describing any steps | |  |  |  |  |
|  |  |  |  |  | to conceal the sequence until | |  |  |  |  |
|  |  |  |  |  | interventions are assigned | |  |  |  |  |
|  | Implementation | | 16c |  | Who will generate the allocation | | - | |  | Page 8 |
|  |  |  |  |  | sequence, who will enrol | |  |  |  |  |
|  |  |  |  |  | participants, and who will assign | |  |  |  |  |
|  |  |  |  |  | participants to interventions | |  |  |  |  |
|  |  | |  |  |  | |  | |  |  |
|  | Blinding | | 17a |  | Who will be blinded after | | - | |  | page 8, 9 |
|  | (masking) | |  |  | assignment to interventions (eg, | |  |  |  |  |
|  |  |  |  |  | trial participants, care providers, | |  |  |  |  |
|  |  |  |  |  | outcome assessors, data | |  |  |  |  |
|  |  |  |  |  | analysts), and how | |  |  |  |  |
|  |  |  | 17b |  | If blinded, circumstances under | | - | |  | Page 8, 9 |
|  |  |  |  |  | which unblinding is permissible, | |  |  |  |  |
|  |  |  |  |  | and procedure for revealing a | |  |  |  |  |
|  |  |  |  |  | participant s allocated inter ention | |  |  |  |  |
|  |  |  |  |  | during the trial | |  |  |  |  |
|  | **Methods: Data** | | **collection,** | **management, and analysis** | | |  |  |  |  |
|  |  |  |  |  |  |  |  |  |  |  |
|  | Data collection | | 18a |  | Plans for assessment and | | - | |  | Page 9 |
|  | methods | |  |  | collection of outcome, baseline, | |  |  |  | Table 1 |
|  |  |  |  |  | and other trial data, including any | |  |  |  | Table 2 |
|  |  |  |  |  | related processes to promote data | |  |  |  |  |
|  |  |  |  |  | quality (eg, duplicate | |  |  |  |  |
|  |  |  |  |  | measurements, training of | |  |  |  |  |
|  |  |  |  |  | assessors) and a description of | |  |  |  |  |
|  |  |  |  |  | study instruments (eg, | |  |  |  |  |
|  |  |  |  |  | questionnaires, laboratory tests) | |  |  |  |  |
|  |  |  |  |  | along with their reliability and | |  |  |  |  |
|  |  |  |  |  | validity, if known. Reference to | |  |  |  |  |
|  |  |  |  |  | where data collection forms can | |  |  |  |  |
|  |  |  |  |  | be found, if not in the protocol | |  |  |  |  |
|  |  |  | 18a.1 |  |  |  |  | Describe what is known about the | | Page 9 |
|  |  |  |  |  |  |  |  | responsiveness of the study | |  |
|  |  |  |  |  |  |  |  | instruments in a population similar to | |  |
|  |  |  |  |  |  |  |  | the study sample | |  |
|  |  |  |  |  |  |  |  |  | |  |
|  |  |  | 18a.2 |  |  |  |  | Describe who will assess the | | Page 8 |
|  |  |  |  |  |  |  |  | outcome (eg, nurse, parent) | |  |
|  |  |  |  |  |  | |  | |  |  |
|  |  |  | 18b |  | Plans to promote participant | | - | |  | Page 9 |
|  |  |  |  |  | retention and complete follow-up, | |  |  |  |  |
|  |  |  |  |  | including list of any outcome data | |  |  |  |  |
|  |  |  |  |  | to be collected for participants | |  |  |  |  |
|  |  |  |  |  | who discontinue or deviate from | |  |  |  |  |
|  |  |  |  |  | intervention protocols | |  |  |  |  |

4

|  | **Section** |  | **Item** |  | **SPIRIT 2013 Item** |  |  | **SPIRIT-Outcomes 2022 item** |  | **Location** |
| --- | --- | --- | --- | --- | --- | --- | --- | --- | --- | --- |
|  |  |  | **No.** |  |  |  |  |  |  | **Reported^b^** |
|  |  |  |  |  |  |  |  |  |  |  |
|  | Data | | 19 |  | Plans for data entry, coding, | | - | |  | Page 9, 10 |
|  | management | |  |  | security, and storage, including | |  |  |  |  |
|  |  |  |  |  | any related processes to promote | |  |  |  |  |
|  |  |  |  |  | data quality (eg, double data | |  |  |  |  |
|  |  |  |  |  | entry; range checks for data | |  |  |  |  |
|  |  |  |  |  | values). Reference to where | |  |  |  |  |
|  |  |  |  |  | details of data management | |  |  |  |  |
|  |  |  |  |  | procedures can be found, if not in | |  |  |  |  |
|  |  |  |  |  | the protocol | |  |  |  |  |
|  | Statistical | | 20a |  | Statistical methods for analysing | | - | |  | Page 10 |
|  | methods | |  |  | primary and secondary outcomes. | |  |  |  |  |
|  |  |  |  |  | Reference to where other details | |  |  |  |  |
|  |  |  |  |  | of the statistical analysis plan can | |  |  |  |  |
|  |  |  |  |  | be found, if not in the protocol | |  |  |  |  |
|  |  |  | 20a.1 |  |  |  |  | Describe any planned methods to | | NA |
|  |  |  |  |  |  |  |  | account for multiplicity in the analysis | |  |
|  |  |  |  |  |  |  |  | or interpretation of the primary and | |  |
|  |  |  |  |  |  |  |  | secondary outcomes (eg, coprimary | |  |
|  |  |  |  |  |  |  |  | outcomes, same outcome assessed | |  |
|  |  |  |  |  |  |  |  | at multiple time points, or subgroup | |  |
|  |  |  |  |  |  |  |  | analyses of an outcome) | |  |
|  |  |  | 20b |  | Methods for any additional | | - | |  | NA |
|  |  |  |  |  | analyses (eg, subgroup and | |  |  |  |  |
|  |  |  |  |  | adjusted analyses) | |  |  |  |  |
|  |  |  | 20c |  | Definition of analysis population | | - | |  | Page 10 |
|  |  |  |  |  | relating to protocol non- | |  |  |  |  |
|  |  |  |  |  | adherence (eg, as randomised | |  |  |  |  |
|  |  |  |  |  | analysis), and any statistical | |  |  |  |  |
|  |  |  |  |  | methods to handle missing data | |  |  |  |  |
|  |  |  |  |  | (eg, multiple imputation) | |  |  |  |  |
|  | **Methods: Monitoring** | | |  |  |  |  |  |  |  |
|  | Data monitoring | | 21a |  | Composition of data monitoring | | - | |  | Page 11 |
|  |  |  |  |  | committee (DMC); summary of its | |  |  |  |  |
|  |  |  |  |  | role and reporting structure; | |  |  |  |  |
|  |  |  |  |  | statement of whether it is | |  |  |  |  |
|  |  |  |  |  | independent from the sponsor | |  |  |  |  |
|  |  |  |  |  | and competing interests; and | |  |  |  |  |
|  |  |  |  |  | reference to where further details | |  |  |  |  |
|  |  |  |  |  | about its charter can be found, if | |  |  |  |  |
|  |  |  |  |  | not in the protocol. Alternatively, | |  |  |  |  |
|  |  |  |  |  | an explanation of why a DMC is | |  |  |  |  |
|  |  |  |  |  | not needed | |  |  |  |  |
|  |  |  | 21b |  | Description of any interim | | - | |  | Page 11 |
|  |  |  |  |  | analyses and stopping guidelines, | |  |  |  |  |
|  |  |  |  |  | including who will have access to | |  |  |  |  |
|  |  |  |  |  | these interim results and make | |  |  |  |  |
|  |  |  |  |  | the final decision to terminate the | |  |  |  |  |
|  |  |  |  |  | trial | |  |  |  |  |
|  | Harms | | 22 |  | Plans for collecting, assessing, | | - | |  | Page 11 |
|  |  |  |  |  | reporting, and managing solicited | |  |  |  |  |
|  |  |  |  |  | and spontaneously reported | |  |  |  |  |
|  |  |  |  |  | adverse events and other | |  |  |  |  |
|  |  |  |  |  | unintended effects of trial | |  |  |  |  |
|  |  |  |  |  | interventions or trial conduct | |  |  |  |  |
|  |  |  |  |  |  |  |  |  |  |  |

5

|  | **Section** |  | **Item** |  | **SPIRIT 2013 Item** |  |  | **SPIRIT-Outcomes 2022 item** |  | **Location** |
| --- | --- | --- | --- | --- | --- | --- | --- | --- | --- | --- |
|  |  |  | **No.** |  |  |  |  |  |  | **Reported^b^** |
|  |  |  |  |  |  |  |  |  |  |  |
|  | Auditing | | 23 |  | Frequency and procedures for | | - | |  | Page 11 |
|  |  |  |  |  | auditing trial conduct, if any, and | |  |  |  |  |
|  |  |  |  |  | whether the process will be | |  |  |  |  |
|  |  |  |  |  | independent from investigators | |  |  |  |  |
|  |  |  |  |  | and the sponsor | |  |  |  |  |
|  | **Ethics and dissemination** | | |  |  |  |  |  |  |  |
|  |  |  |  |  |  |  |  |  |  |  |
|  | Research ethics | | 24 |  | Plans for seeking research ethics | | - | |  | Page 11 |
|  | approval | |  |  | committee/institutional review | |  |  |  |  |
|  |  |  |  |  | board (REC/IRB) approval | |  |  |  |  |
|  | Protocol | | 25 |  | Plans for communicating | | - | |  | Page 11, 12 |
|  | amendments | |  |  | important protocol modifications | |  |  |  |  |
|  |  |  |  |  | (eg, changes to eligibility criteria, | |  |  |  |  |
|  |  |  |  |  | outcomes, analyses) to relevant | |  |  |  |  |
|  |  |  |  |  | parties (eg, investigators, | |  |  |  |  |
|  |  |  |  |  | REC/IRBs, trial participants, trial | |  |  |  |  |
|  |  |  |  |  | registries, journals, regulators) | |  |  |  |  |
|  | Consent or | | 26a |  | Who will obtain informed consent | | - | |  | Page 7, 11, 12 |
|  | assent | |  |  | or assent from potential trial | |  |  |  |  |
|  |  |  |  |  | participants or authorised | |  |  |  |  |
|  |  |  |  |  | surrogates, and how (see Item | |  |  |  |  |
|  |  |  |  | 32) | |  |  |  |  |  |
|  |  |  | 26b |  | Additional consent provisions for | | - | |  | Page, 7, 11, 12 |
|  |  |  |  |  | collection and use of participant | |  |  |  |  |
|  |  |  |  |  | data and biological specimens in | |  |  |  |  |
|  |  |  |  |  | ancillary studies, if applicable | |  |  |  |  |
|  | Confidentiality | | 27 |  | How personal information about | | - | |  | Page 9, 10, 11, |
|  |  |  |  |  | potential and enrolled participants | |  |  |  | 12 |
|  |  |  |  |  | will be collected, shared, and | |  |  |  |  |
|  |  |  |  |  | maintained in order to protect | |  |  |  |  |
|  |  |  |  |  | confidentiality before, during, and | |  |  |  |  |
|  |  |  |  |  | after the trial | |  |  |  |  |
|  | Declaration of | | 28 |  | Financial and other competing | | - | |  | Page 14 |
|  | interests | |  |  | interests for principal investigators | |  |  |  |  |
|  |  |  |  |  | for the overall trial and each study | |  |  |  |  |
|  |  |  |  |  | site | |  |  |  |  |
|  | Access to data | | 29 |  | Statement of who will have | | - | |  | Page 12 |
|  |  |  |  |  | access to the final trial dataset, | |  |  |  |  |
|  |  |  |  |  | and disclosure of contractual | |  |  |  |  |
|  |  |  |  |  | agreements that limit such access | |  |  |  |  |
|  |  |  |  |  | for investigators | |  |  |  |  |
|  | Ancillary and | | 30 |  | Provisions, if any, for ancillary and | | - | |  | Page 11 |
|  | post-trial care | |  |  | post-trial care, and for | |  |  |  |  |
|  |  |  |  |  | compensation to those who suffer | |  |  |  |  |
|  |  |  |  |  | harm from trial participation | |  |  |  |  |
|  | Dissemination | | 31a |  | Plans for investigators and | | - | |  | Page 12 |
|  | policy | |  |  | sponsor to communicate trial | |  |  |  |  |
|  |  |  |  |  | results to participants, healthcare | |  |  |  |  |
|  |  |  |  |  | professionals, the public, and | |  |  |  |  |
|  |  |  |  |  | other relevant groups (eg, via | |  |  |  |  |
|  |  |  |  |  | publication, reporting in results | |  |  |  |  |
|  |  |  |  |  | databases, or other data sharing | |  |  |  |  |
|  |  |  |  |  | arrangements), including any | |  |  |  |  |
|  |  |  |  |  | publication restrictions | |  |  |  |  |
|  |  |  | 31b |  | Authorship eligibility guidelines | | - | |  | Page 12 |
|  |  |  |  |  | and any intended use of | |  |  |  |  |
|  |  |  |  |  | professional writers | |  |  |  |  |

6

|  | **Section** |  | **Item** |  | **SPIRIT 2013 Item** |  |  | **SPIRIT-Outcomes 2022 item** |  | **Location** |
| --- | --- | --- | --- | --- | --- | --- | --- | --- | --- | --- |
|  |  |  | **No.** |  |  |  |  |  |  | **Reported^b^** |
|  |  |  |  |  |  |  |  |  |  |  |
|  |  |  | 31c |  | Plans, if any, for granting public | | - | |  | Page 12 |
|  |  |  |  |  | access to the full protocol, | |  |  |  |  |
|  |  |  |  |  | participant-level dataset, and | |  |  |  |  |
|  |  |  |  |  | statistical code | |  |  |  |  |
|  | **Appendices** | |  |  |  |  |  |  |  |  |
|  | Informed | | 32 |  | Model consent form and other | | - | |  | Appendix |
|  | consent | |  |  | related documentation given to | |  |  |  |  |
|  | materials | |  |  | participants and authorised | |  |  |  |  |
|  |  |  |  |  | surrogates | |  |  |  |  |
|  | Biological | | 33 |  | Plans for collection, laboratory | | - | |  | Appendix |
|  | specimens | |  |  | evaluation, and storage of | |  |  |  |  |
|  |  |  |  |  | biological specimens for genetic | |  |  |  |  |
|  |  |  |  |  | or molecular analysis in the | |  |  |  |  |
|  |  |  |  |  | current trial and for future use in | |  |  |  |  |
|  |  |  |  |  | ancillary studies, if applicable | |  |  |  |  |

^a^It is strongly recommended that this checklist be read in conjunction with the SPIRIT (Standard Protocol Items: Recommendations for Interventional Trials) Statement paper for important clarification on the items. Amendments to the protocol should be tracked and dated. The SPIRIT checklist is cop righted b the SPIRIT Gro p nder the Creati e Commons Attrib tion-NonCommercial-NoDeri s 3.0 Unported license and is reprod ced ith permission.

^b^Indicates page numbers and/or manuscript location: to be completed by authors.

Please cite as: Butcher NJ, Monsour A, Mew EJ, et al. Guidelines for reporting outcomes in trial protocols: the SPIRIT-Outcomes 2022

7

extension. JAMA. Published online December 13, 2022. doi:10.1001/jama.2022.21243
